# Relative rate and location of intra-host HIV evolution to evade cellular immunity are predictable

**DOI:** 10.1038/ncomms11660

**Published:** 2016-05-23

**Authors:** John P. Barton, Nilu Goonetilleke, Thomas C. Butler, Bruce D. Walker, Andrew J. McMichael, Arup K. Chakraborty

**Affiliations:** 1 Ragon Institute of MGH, MIT and Harvard, Cambridge, Massachusetts 02139, USA; 2 Department of Chemical Engineering, Massachusetts Institute of Technology, Cambridge, Massachusetts 02139, USA; 3 Department of Physics, Massachusetts Institute of Technology, Cambridge, Massachusetts 02139, USA; 4 Institute for Medical Engineering and Science, Massachusetts Institute of Technology, Cambridge, Massachusetts 02139, USA; 5 Department of Microbiology, Immunology and Medicine, University of North Carolina, Chapel Hill, North Carolina 27599, USA; 6 Nuffield Department of Medicine, University of Oxford, Old Road Campus, Headington, Oxford OX3 7FZ, UK; 7 Howard Hughes Medical Institute, Chevy Chase, Maryland 20815, USA; 8 Department of Chemistry, Massachusetts Institute of Technology, Cambridge, Massachusetts 02139, USA; 9 Department of Biological Engineering, Massachusetts Institute of Technology, Cambridge, Massachusetts 02139, USA

## Abstract

Human immunodeficiency virus (HIV) evolves within infected persons to escape being destroyed by the host immune system, thereby preventing effective immune control of infection. Here, we combine methods from evolutionary dynamics and statistical physics to simulate *in vivo* HIV sequence evolution, predicting the relative rate of escape and the location of escape mutations in response to T-cell-mediated immune pressure in a cohort of 17 persons with acute HIV infection. Predicted and clinically observed times to escape immune responses agree well, and we show that the mutational pathways to escape depend on the viral sequence background due to epistatic interactions. The ability to predict escape pathways and the duration over which control is maintained by specific immune responses open the door to rational design of immunotherapeutic strategies that might enable long-term control of HIV infection. Our approach enables intra-host evolution of a human pathogen to be predicted in a probabilistic framework.

## Abstract (web summary)

HIV evolves within infected persons to escape being destroyed by the immune system. Here, Barton *et al*. combine evolutionary dynamics and statistical physics to simulate this process, successfully predicting the relative rate and location of escape mutations in viral sequences for a cohort of HIV-infected persons.

## Graphical abstract

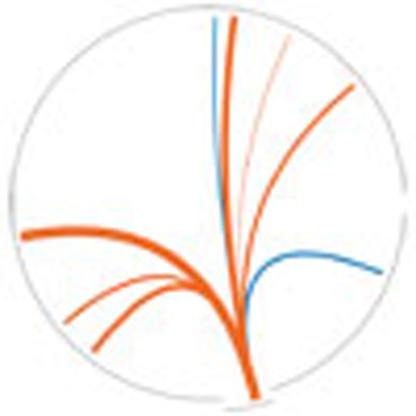

HIV evolves to accumulate mutations that enable the virus to escape host immunity^1^, limiting control of infection^2^,^3^. Viral fitness constraints limit these mutational pathways^4^,^5^,^6^, but these constraints are complicated because the fitness cost of escape mutations can be compensated by mutations elsewhere in the proteome^7^,^8^. This can make the ability to escape immune responses by mutation contingent on the virus's sequence background. Therefore, simply focusing immune responses on parts of the viral proteome that appear conserved by local measures of mutability (for example, entropy) is insufficient for the design of effective strategies for controlling infection by limiting escape^6^,^9^,^10^,^11^.

Ideally, vaccine-induced immune responses should be directed towards combinations of epitopes where escape mutations are highly deleterious in diverse sequence backgrounds, thus minimizing the probability of escape and allowing long-term control of infection. Indeed, prior studies have observed connections between epitope targeting and disease progression^12^. To take steps towards this goal, knowledge of how the virus's replicative fitness depends on its sequence (its fitness landscape), with explicit accounting for coupling between multiple mutations, is required. This knowledge, combined with evolutionary dynamics, can then predict how diverse viral strains will evolve in individuals when subjected to different immune responses. To our knowledge, such studies of evolutionary dynamics have not been performed previously for any human pathogen, but could be used to discover optimal combinations of epitopes as vaccine targets.

Recently, we proposed a computational model to translate sequence data of HIV polyproteins into estimates of how the frequency of different HIV strains across the host population depends on genetic sequence^10^,^13^. This least-biased^14^, or maximum-entropy, model for the prevalence is constrained to reproduce the frequency of single and double mutations observed in the HIV sequence data, and takes the form of a Potts model from statistical physics. Similar maximum-entropy models have been used to study the properties of neuronal networks^15^, segments of antibody sequences^15^,^16^ and structural contacts in protein families^17^.

Following simple evolutionary models, fitter viruses are expected to be more prevalent, at least over very long time scales (that is, in the limit that the distribution of sequences reaches a steady state)^18^,^19^. The connection between prevalence and fitness could be obscured by many factors, including the breaking of this assumption, especially when the virus population is under the influence of host immunity, which drives the evolution of escape mutations. However, for the HIV population, past analyses and the arguments below suggest that the relationship between prevalence and fitness is relatively simple.

Although human T-cell responses lead to the selection of escape mutants, these responses are extraordinarily diverse^20^, because of the enormous diversity of HLA genes in the population. Thus, the same epitopes are not consistently targeted among different hosts. For example, of the 363 residues in the immunogenic proteins p17 and p24, only 46 are targeted by >10% of humans, none by >23% and 146 residues are not targeted at all^10^. Furthermore, deleterious escape mutations can revert when the virus is transmitted to a new host^21^. Although a few HLA-epitope combinations have been associated with better outcome in infected persons, HIV has not been persistently subjected to classes of effective natural or vaccine-induced memory immune responses. Thus, unlike viruses such as influenza^22^,^23^, at the population level, HIV evolution is not narrowly directed over time because of the progressive fixation of mutations to evade memory immune responses.

Of course, in individual hosts the virus evolves to evade host immunity and this is an important driver forcing HIV to explore sequence space. Compensatory mutations can arise in conjunction with deleterious escape mutations, and therefore these combinations of mutations are observed more frequently than by chance in the circulating virus population. Similarly, combinations of mutations that are especially deleterious may be observed less frequently than by chance. These correlations, which reflect the host–pathogen *riposte*, are the key inputs to our inference procedure, and thus our landscape describes the collective mutational pathways that HIV uses to evade host immunity. Because of the great diversity of human immune responses, specific sets of correlated mutations observed at the population level, which inform our inference procedure, cannot be uniquely assigned to individual HLA molecules alone^24^.

Theoretical and computational studies suggest that, for the reasons noted above, the rank order of the inferred prevalence of HIV strains is statistically similar to the rank order of intrinsic fitness^25^. The same analysis suggests that phylogeny, which biases the sequence distribution due to shared evolutionary history, also affects the relationship between prevalence and fitness. These effects are small, however, unless the sequences are separated by many mutations. Viral sequences that evolve in a single infected individual are more closely related. The arguments noted above suggest that the HIV population is approximately at a steady state for strains separated by modest mutational distances, and thus our inferred landscape can be used to study HIV evolution in patients. Recent work also suggests that recombination facilitates our inference of fitness landscapes of HIV from virus population data^26^. Moreover, experimental tests showed robust correlation between our fitness estimates for HIV Gag p17 and p24 and *in vitro* replicative capacity for a library of HIV strains generated by introducing mutations into these subunit proteins of an NL4-3 reference strain^10^,^13^. These results support the assertion that prevalence and fitness should be closely linked for HIV, at least for sequences that are phylogenetically not too distant.

Here, we first infer the fitness/prevalence landscape of HIV polyproteins. We then combine the inferred fitness landscape with a simple model from population genetics, and incorporate knowledge of the host immune response to investigate how fitness constraints influence *in vivo* non-equilibrium viral evolution in response to T-cell-mediated immune pressure in a cohort of 17 persons during acute HIV infection. These simulations yield predictions for both the relative time necessary for specific CD8^+^ T-cell epitope escape mutants to dominate the virus population in the host as well as the specific residues at which escape mutations are most likely to arise. We illustrate the potential effects of the viral sequence background on escape through some examples. Explicit simulation of dynamical escape trajectories takes into account the contribution of multiple pathways to escape, and we contrast the enhanced predictive power of the dynamic simulations with static measures of fitness. Our results suggest that by combining stochastic evolutionary dynamics with the fitness landscape of a human virus and knowledge of the immune response, its evolution in individual hosts is predictable.

## Results

### Fitness landscape and patient data

In our model, the prevalence/fitness *P*(**z**) of an HIV protein sequence **z**={*z*_1_, *z*_2_, …, *z*_*N*_} is


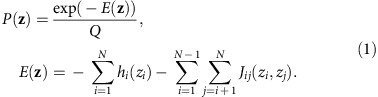


Here, *N* is the length of the sequence and *Q* is a normalizing factor; *z*_*i*_ denotes the amino acid at each residue *i*. Following the language of statistical physics, our proxy for fitness is a quantity referred to as energy (*E*). The energy depends on the mutability of individual residues (quantified by the *h* parameters) and the entire sequence of the viral protein with explicit account for synergistic (or antagonistic) interactions between mutations in different residues, quantified by the *J* parameters. Sequences with high energies are estimated to be relatively unfit, and vice versa.

Using equation (1) we inferred the fitness landscapes of all HIV proteins except gp120, far beyond the limited set of proteins we had previously considered^10^,^13^, on the basis of the HIV sequence data obtained from thousands of infected individuals beyond the patients studied in this paper (Supplementary Table 1). The exclusion of gp120 was due to the combination of its length and high variability, which makes model inference more challenging. We note that, although the inference method^27^,^28^ (see the ‘Methods' section for details) constrains only the frequencies of single and double mutations to be those observed in the sequence data, the probabilities of observing higher-order mutations in the sequences are also recovered (Supplementary Fig. 1).

In the infected individuals that we studied, a comprehensive analysis of acute-/early-phase CD8^+^ T-cell responses to autologous virus had been performed and time to escape had been experimentally defined^29^. Because of the likely importance of early T-cell responses in disease progression^2^,^30^,^31^, we focused on epitopes targeted early in infection (first response to the epitope detected ≤50 days post estimated Fiebig stage I/II (ref. 32), spanning a time from the first detection of plasma viremia to shortly after resolution of peak viremia in acute infection). Data were also collected most frequently during acute infection^29^, allowing for a more accurate estimation of early escape times^33^ (see the ‘Methods' section).

### Illustrations of the importance of sequence background on escape

**Figure 1:**
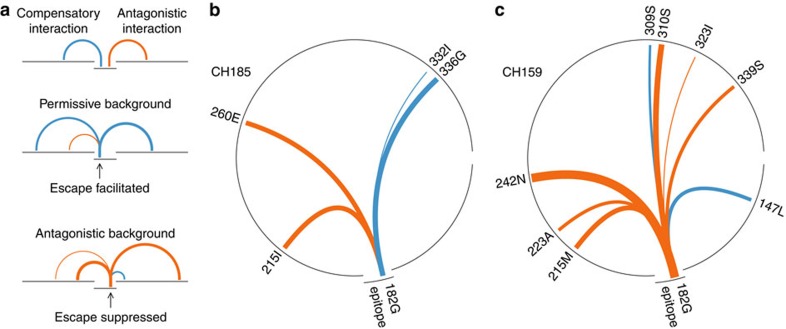
Specific residues in the sequence background can strongly influence the time to escape. (**a**) In general, escape may occur more rapidly on permissive sequence backgrounds having many compensatory interactions with potential escape mutations. Escape may also be delayed or can occur at other sites when strong antagonistic interactions increase the fitness cost of certain escape mutations. (**b**,**c**) Strong interactions between the Gag TL9 epitope escape mutation 182 G and the transmitted/founder sequence background in patients CH185 (**b**) (escape time=122 days) and CH159 (**c**) (escape time>1,103 days) differ significantly. All strong interactions (|*J*|>0.1, see equation (1)) between 182 G and the p24 protein sequence background, represented by the circles, are shown, with the width of the link proportional to the magnitude of the coupling. Compensatory or synergistic interactions (*J*>0) lower the fitness cost of mutation, thus promoting escape. Antagonistic interactions (*J*<0) increase the fitness cost of mutation, discouraging escape.

Cases of identical epitopes targeted by different patients illustrate how sequence background can strongly affect the dynamics of escape. As one example, escape from the Gag epitope TPQDLNTML_180–188_ (TL9) occurred after 122 days in patient CH185, but in patient CH159, who targeted this same epitope restricted by the same class I molecule, escape mutations were not observed even up to 1,103 days after the response to this epitope was first detected. Our calculations show (Fig. 1a,b; see also Table 1) that this is because of differences in the sequence background in the transmitted/founder (T/F) viral strains in these two patients. For patient CH185, the background amino acid sequence was far more conducive to escape. In contrast, specific amino acids in the sequence background in patient CH159 displayed strong antagonistic interactions with the escape mutation 182 G (that is, large negative *J* parameters, see equation (1)), thus substantially increasing the predicted fitness cost of mutations within this epitope for CH159.

As another example of the effects of differences in background viral sequence on escape times, consider the Gag epitope TSTLQEQVAW_240–249_ (TW10) targeted by patients CAP239 and CH198. In CAP239 escape occurred in just days, even though TW10 was considered to be a protective epitope^12^ where escape often incurs a high fitness cost^7^. The Shannon entropy of this epitope, a quantity that can correlate with time to escape^29^, is also fairly low (*S*=0.19, in the bottom 31% of epitope entropies), making the rapid escape appear puzzling. However, the average fitness cost for mutational escape (*ΔE*, see the ‘Methods' section) for this epitope, which includes the effects of the sequence background, is very low (*ΔE*=−1.4, in the bottom 15% of all epitopes considered here). This is true in part because the T/F virus in patient CAP239 contained the mutations H219Q and I223V, compared with the consensus sequence (see ref. 29), which are known to partially compensate for the fitness cost of escape mutation in the TW10 epitope^8^. In our model these residues had a synergistic interaction with the observed escape mutations T242N and A248T (see Supplementary Fig. 2a,b), contributing to the low value of *ΔE*. Thus, the model successfully predicts rapid escape, whereas Shannon entropy measures do not. The sequence background of patient CH198 also contained specific amino acids that compensated for the eventual T242N escape mutation, which arose after 220 days, but mutations at residues like A248T were suppressed by other residues with antagonistic interactions (Supplementary Fig. 2c,d). This resulted in a higher estimated fitness cost of escape (*ΔE*=0.1). Thus, we predict that escape should occur more slowly in patient CH198, and only through the T242N mutation, in agreement with the clinical data.

**Figure 2:**
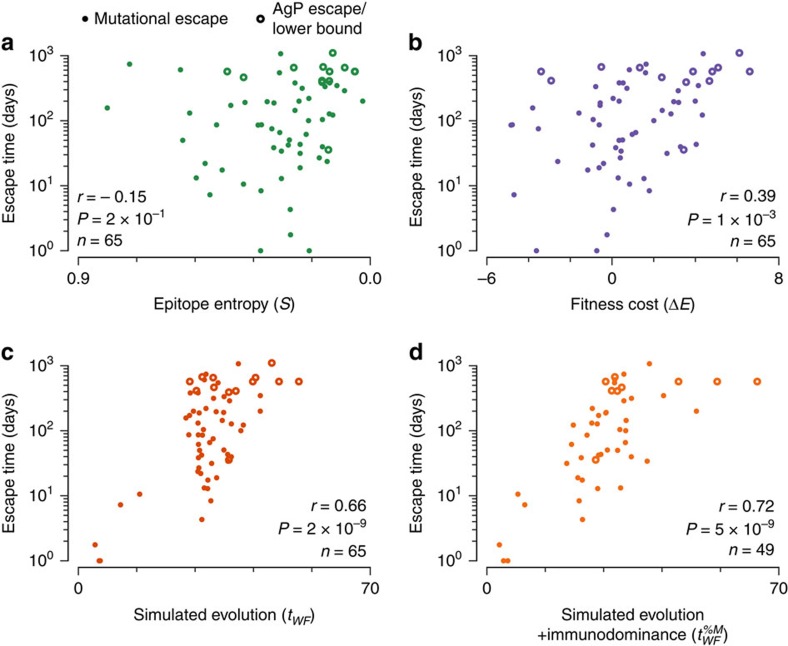
Measured escape times are strongly correlated with the simulated escape time. Compared with the epitope entropy (**a**) and the average fitness cost of escape mutations (**b**), the time to escape in evolutionary simulation (**c**) shows more robust correlation with the escape time inferred from clinical data. When escape occurred through AgP mutations affecting presentation of the epitope (open circles), the time at which AgP mutants dominate the population is substituted as a lower bound for the escape time (*n*=3 cases). Similarly, the final time at which sequence samples were collected was substituted as a lower bound on the escape time when no escape was observed (*n*=10). (**d**) Information about immunodominance can be incorporated into evolutionary simulations, improving the predicted escape times for epitopes where this information is available (*n*=49). In all cases, epitopes where escape was observed at the time when T-cell response was detected are excluded (*n*=6 total, out of which 4 have immunodominance measurements). Epitopes studied include those derived from all HIV proteins except Vpu (because no patients targeted epitopes in Vpu early in infection) and gp120.

The effects of epistatic interactions on escape will not always be as marked as for the cases discussed above. But their importance in general is indicated by the fact that, using our fitness landscape model alone, which considers the entire protein, the average fitness cost we estimate is more strongly correlated with the observed escape time for each epitope (Pearson's *r*=0.39, *P*=1 × 10^−3^, *n*=65, see Fig. 2b) than the average Shannon entropy (*S*) of residues in the reactive 8–12-amino-acid (aa) epitope (Pearson's *r*=−0.15, *P*=2 × 10^−1^, Fig. 2a, studied in connection with escape in ref. 29). However, fitness cost alone cannot predict the time to escape because such a static measure does not account for the stochastic dynamics of virus evolution and multiple escape pathways that may become available, nor does it incorporate the effects of sequence heterogeneity in the evolving swarm of viruses. Indeed, we observe that the number of residues in each epitope with low-energy (*E*<2) mutations available is also significantly correlated with time to escape (Pearson's *r*=−0.32, *P*=6 × 10^−3^), hinting at the potential importance of multiple escape paths (see Supplementary Fig. 3 for further details). In addition, the static approach does not accommodate the strength of the T-cell response to each epitope.

### Predicting relative escape times through evolutionary dynamics

We simulated the evolution of the virus population in response to CD8^+^ T-cell-mediated immune pressure on specific epitopes using a Wright–Fisher-like model from population genetics. The model describes evolution through discrete rounds of replication, mutation and selection (see the ‘Methods' section). Mapping from energy values to differences in fitness was estimated using measurements of HIV replication *in vitro* obtained from a separate study^13^ (see Supplementary Fig. 4). We used the sample of viral sequences obtained at the time the T-cell response was first detected as the starting population for the simulation. This allows us to consider the effects of diverse viral sequence backgrounds on escape. To capture the effects of the ongoing killing of infected cells by T cells specific for the targeted epitope, all sequences without non-synonymous mutations in the epitope had their fitness reduced by a fixed amount, chosen large enough so that escape conferred a selective advantage (for details, see the ‘Methods' section).

For each epitope studied, we carried out many simulations and computed the mean number of discrete evolutionary generations (*t*_WF_) that elapsed before escape mutants comprised >50% of the total virus population. The values of *t*_WF_ can be interpreted as relative rates for the evolution of escape mutants for each epitope. The values of *t*_WF_ are strongly correlated with the true escape times observed in the patients (Pearson's *r*=0.66, *P*=2 × 10^−9^, Fig. 2c), vastly improving predictions based on Shannon entropy or static fitness cost estimates alone. In these calculations we excluded 6 epitopes where the fraction of escape mutants in the virus population at the time point when the T-cell response was initially detected was ≥50%. If these data points are included, the correlation between *t*_WF_ and the true escape time becomes even stronger (Pearson's *r*=0.81, *P*=2 × 10^−17^, Supplementary Fig. 5, including error bars on true and simulated escape times). This is because we predict that escape occurs very rapidly in these cases (see the ‘Methods' section and ref. 29). It is important to note that the founder viruses in the patients where escape mutations in the six epitopes were >50% of the quasispecies at the first time point of observation did not contain these escape mutations. So, we have excluded these cases from statistical analyses by an abundance of caution only.

The characteristics of the immune response directed towards each epitope also influence the process of escape. In particular, stronger immune responses will result in a greater selective advantage for the virus to evolve a mutation in a targeted epitope to evade the immune response. The balance between this selective advantage and the intrinsic fitness cost incurred by making the mutation determines the location and kinetics of evolution of escape mutations. The relative strength of the immune response targeting epitopes (immunodominance) and the incurred intrinsic fitness costs are independent effects. The larger the intrinsic fitness costs incurred by making a mutation, the greater must be the strength of the immune response directed towards the corresponding epitope in order for the virus to evolve an escape mutation at that residue. Immunodominance information alone provides no knowledge about which regions of the virus should be targeted by vaccine-induced immune responses to minimize the rate of escape due to large fitness costs. Given the same strength of the immune response directed towards two epitopes, escape will be faster in the epitope for which the fitness cost of evolving a mutation (given the sequence background) is lower. The intrinsic fitness cost can be estimated more accurately using our methods compared with past efforts using entropy.

In this clinical data set, vertical immunodominance, the fraction of the total measured HIV-1-specific T-cell response directed towards a specific epitope (%*M*), was determined for 53 epitopes^29^. To obtain the best predictions of escape, this information about the strength of T-cell responses should be combined with estimates of viral fitness. Immunodominance can naturally be incorporated into our Wright–Fisher simulations by increasing the fitness penalty for viruses without escape mutations in proportion with the strength of the immune response directed towards each epitope (*t*_WF_^%M^). This further improves our ability to predict escape times for cases where immunodominance information is known (Pearson's *r*=0.72, *P*=5 × 10^−9^, Fig. 2d; see Supplementary Table 2 for comparisons in rank correlations). Note that it was previously found that immunodominance by itself correlated with time to escape (Pearson's *r*=−0.41, *P*=2 × 10^−3^, for the subset of *n*=53 epitopes for which immunodominance information is available^29^). By combining the two forces at play in the evolution of escape mutations—fitness costs and strength of immune responses—the ability to predict time to escape improves significantly.

Next we further quantified the relative statistical power of each predictor of escape time. To obtain a more sensitive measure of contributions to the escape time we used a Cox proportional hazards (CPH) model, which properly accounts for whether or not escape was observed for each epitope during the time of observation (Table 2). Here, we found that the predictive power of the time to escape in Wright–Fisher simulations without including immunodominance information (*t*_WF_; pseudo-*R*^2^=0.37, *P*=9 × 10^−6^, *n*=49) markedly improves upon both the static fitness cost (*ΔE*; pseudo-*R*^2^=0.10, *P*=0.02) and epitope entropy (*S*; pseudo-*R*^2^=0.05, *P*=0.11), even when rapidly escaping epitopes are excluded. Overall, *t*_WF_ displays similar predictive power to %*M* (pseudo-*R*^2^=0.33, *P*=5 × 10^−5^), suggesting that both viral and host factors strongly influence the rate of escape. Encouragingly, we found that simulations combining our inferred fitness landscape with knowledge of immunodominance patterns (*t*_WF_^%M^; pseudo-*R*^2^=0.53, *P*=1 × 10^−7^) capture much of the predictive power of both variables summed individually (pseudo-*R*^2^=0.56). This result is consistent with our argument above that the intrinsic fitness cost of escape mutations and the corresponding selective advantage due to immune evasion are independent effects whose balance determines the kinetics of escape. These results also hold in patient-stratified CPH models, which incorporate patient-specific baseline escape rates (Supplementary Table 3). Overall we found a consistent hierarchy in which the Wright–Fisher simulations including immunodominance have by far the greatest predictive power, followed by *t*_WF_ and %*M* separately, then by static fitness costs and finally by the epitope entropy *S*.

### Dynamical predictions of the residues where escape occurs

**Figure 3:**
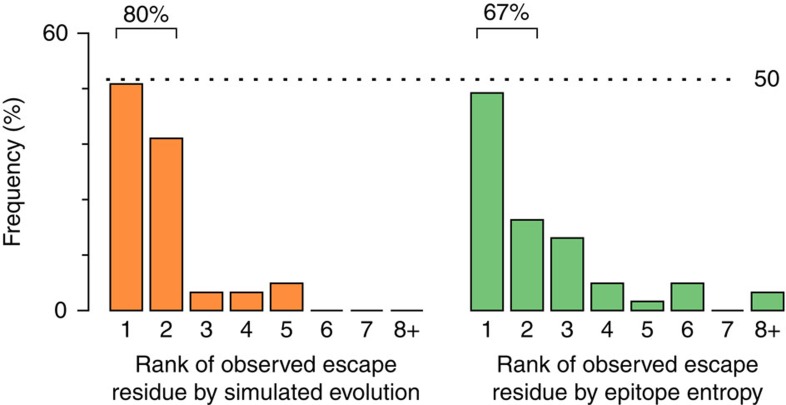
Simulation of evolution with the fitness landscape enhances prediction of the residues at which escape mutations occur. In the great majority of epitopes (*n*=51), the most commonly observed location of escape mutations in the clinical data at the time that escape mutants first comprise >50% of the virus population corresponds to one of the two top residues where mutations are most frequently observed in simulated evolution (44/51=86%). For comparison, the residue where escape mutations are observed most often has one of the top two highest Shannon entropies in 34/51=67% of cases. Epitopes where escape was observed at the time the T-cell response was detected are excluded (*n*=6), as is one epitope without detailed escape sequence data.

Following the hypothesis that escape mutations should preferentially appear at residues where the fitness cost of mutation is minimized, the same methods described above can also be used to predict the residues where escape mutations are most likely to emerge. For each targeted epitope, we ordered each residue in the epitope according to how often an escape mutation was observed at that residue in simulations of evolutionary dynamics (from high to low). We then counted the frequency of escape mutations observed at each residue in the clinical data at the time that escape mutants first comprised ≥50% of the virus population. Figure 3 shows that in the great majority of epitopes (86%) the most common residue where escape mutations arose in patients is one of the top two predicted residues. For reference, these results are compared with predictions based on epitope entropy, where it is assumed that escape mutations are more likely to occur at residues with higher entropy (67% of escapes occur at sites within the top two highest entropies). Similar results are also obtained for the prediction of the most common residue at which escape mutations are observed through the entire time course of in-host virus evolution (Supplementary Fig. 6, see Supplementary Fig. 7 for further detailed results).

## Discussion

Our results show that the relative time to escape from HIV-specific CD8^+^ T-cell responses, as well as the location of emerging escape mutations, is predictable *in silico*, given knowledge of the epitopes targeted by CD8^+^ T cells and the infecting virus's sequence. Collectively, our results emphasize the importance of viral factors in the kinetics and location of escape from T-cell-mediated immune control in early HIV infection when virus set point is being established, and reveal predictable constraints on HIV evolution.

Recent work has also highlighted the role of viral fitness in HIV transmission, observing a significant bias towards the transmission of fitter viruses over less fit variants^34^. Thus, it is especially important to identify epitopes, or combinations of epitopes, where escape exacts a high fitness cost in diverse sequence backgrounds, because targeting of these epitopes through vaccination could not only lead to control of viral loads to low levels but potentially also to reduced replicative fitness of patient virus populations. This effect could result in further reduction in transmission even beyond the benefits of controlling infection in individual patients^34^. Identification of combinations of epitopes where simultaneous mutations are deleterious requires knowledge of the large antagonistic epistatic interactions. This is especially true given that, for many epitopes, it appears that multiple potential escape pathways with similar fitness costs exist (see Supplementary Fig. 3). Moreover, the ability to make accurate predictions of escape pathways should have implications for defining optimal targeting of immune responses capable of controlling virus activated from the virus reservoir^35^, with implications for immunotherapeutic interventions to effect a functional cure.

We note that the evolutionary dynamics considered here incorporate several simplifying assumptions. First, we treat the effective population size as constant, a reasonable assumption in the chronic phase when viral load is fairly stable. Variable population sizes may lead to a better description of escape in the acute phase, when viral load is dynamic, but the appropriate relationship between viral load and model-specific effective population size is unclear. Second, we have conservatively assumed that any non-synonymous mutation within a targeted epitope confers escape. It is not certain that all mutations impair T-cell recognition (the published data are somewhat conflicting^36^,^37^,^38^,^39^), but the majority probably do. As it is impossible to know in general which mutations would lead to abrogation of recognition, the only well-controlled approximation that we are aware of is the one we have used. Detailed knowledge of how individual mutations affect epitope-HLA binding and CD8^+^ T-cell recognition would help to improve the results we have shown here by identifying the specific mutations that effectively confer escape. Such differing effects of mutations within reactive epitopes is another reason that considering multiple escape pathways is important: the existence of several escape pathways with low intrinsic fitness costs could allow the virus to select for escape mutants with higher effective fitness through decreased recognition by the host immune system. More realistic simulations should also include a time-varying fitness penalty for viruses without escape mutations, to take into account the dynamic growth and contraction of epitope-specific CTL (cytotoxic T lymphocyte) clones. Despite these simplifications, our results show good agreement, and significant enhancement over Shannon entropy alone, with relative rates of escape *in vivo* as well as the identity of residues where escape mutations arise. Future refinements are expected to further improve the ability to predict HIV evolution in patients.

Here we carried out Wright–Fisher simulations with and without recombination at the level of single proteins, finding comparable results in each case. This may be because the donors in this cohort were all infected by a single T/F virus, and so escape by recombination without new mutations would not be possible (in cases of multiple infection such escapes can occur^40^). Recombination may be an important feature, however, in extended models including whole-genome evolution, or in cases of multiple infection.

Recent work has also shown the potential importance of clonal interference in the kinetics of escape^11^. Our simulations include the possibility of clonal interference between competing escape variants for the same epitope, but they do not currently take into account competition between sequences with escape mutations in different epitopes. Clonal interference should lead to greater uncertainty in escape times as stochastic effects become more important; however, it should not affect the typical ordering of escape mutations. This is because the same escape mutations can arise on any sequence background, and barring intergenic epistatic effects (which have been estimated to be low in previous studies^9^), on average, escape should occur more rapidly at epitopes where the fitness cost of mutation is minimal. However, the incorporation of clonal interference effects may be important in future more detailed models of viral evolution to most accurately capture times to escape and their statistical uncertainties.

While we have focused on T cells, the methods we have detailed here are not limited to this case alone. Similar approaches could be used to determine whether certain combinations of broadly neutralizing antibody responses are most likely to target nonlinear epitopes to effectively control viral loads to low levels for long times, for example.

## Methods

### Patient cohort

The cohort comprised 17 subjects (10 male and 7 female) identified in acute HIV-1 infection (Fiebig stages I–IV) recruited under the CHAVI 001 and CAPRISA studies at sites in the United States, Malawi and South Africa^29^. US subjects were infected with clade B viruses, whereas all African subjects were infected with clade C viruses. Candidate epitopes in reactive 18 mers that previously could not be reliably identified were selected according to the criteria in Supplementary Table 4 (details are given in the subsection ‘Epitope identification').

### Sequence data for the Potts model

We downloaded multiple sequence alignments (MSA) of HIV-1 clade B and clade C protein sequences from the Los Alamos National Laboratory HIV sequence database (www.hiv.lanl.gov; accessed 6th October 2014). The MSA were then processed to remove insertions relative to the HXB2 reference sequence (GenBank accession code K03455). To improve sequence quality, sequences labelled as ‘problematic' in the sequence database were not downloaded, and sequences with gaps or ambiguous amino acids present at >5% of residues were removed from the MSA. The remaining ambiguous amino acids were imputed with simple mean imputation. For details on the number of sequences obtained for each protein/clade, see Supplementary Table 1.

Each sequence in the MSA can be represented as a vector of variables ***z***={*z*_1_, z_2_, …, *z*_*N*_}, *z*_*i*_∈{A, R, …, V, −}, where *N* is the length of the protein sequence. Each of the *z*_*i*_ represents the amino acid (or gap) present at residue *i* in the sequence. We refer to possible values of the *z*_*i*_ as states. Our goal will be to infer a model that accurately describes the distribution of HIV sequences circulating in the population, of which the sequences in the MSA are a sample. To describe this distribution we focus on the lowest moments: the frequency of each state at each residue, and the frequency of each pair of states at each pair of residues. These are given by


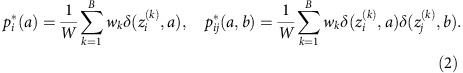


Here *k* is an index running from 1 to *B* used to label each sequence in the MSA, and *B* is the total number of sequences in the MSA. The function *δ* is the Kronecker *δ* function,


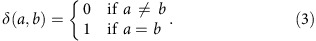


To prevent multiple sequences obtained from the same individual from biasing the sequence distribution, we weight the contribution of each sequence labelled *k* in the MSA by a factor *w*_*k*_. We set *w*_*k*_ equal to one divided by the total number of sequences in the MSA obtained from the same individual from whom the sequence labelled *k* was extracted. In this way, the total weight of the sequences from each individual is equal. The normalizing factor *W* in equation (2) is the number of unique individuals from whom the sequences in the MSA were obtained, given equivalently by *W*=∑_*k*_*w*_*k*_. Following standard terminology in statistical physics, we refer to the *p*_i_*(*a*) and *p*_ij_*(*a*,*b*) given in equation (2) as correlations.

### Maximum entropy inference

There are, in principle, a vast family of probabilistic models that could reproduce the correlations observed in equation (2). The ‘least biased' model capable of reproducing the observed correlations, defined as the model that maximizes the entropy of the sequence distribution, is the Potts model, in which the probability of observing a particular sequence **z** is


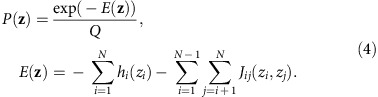


Here *E*(**z**) is referred to as the energy of the sequence **z**, and


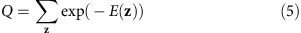


is a normalizing factor ensuring that the probabilities of all sequences sum to one. The sum in equation (5) is over all sequences of length *N*.

The parameters *h*_*i*_(*a*)*, J*_*ij*_(*a*,*b*) in equation (4) are to be chosen such that the Potts model correlations


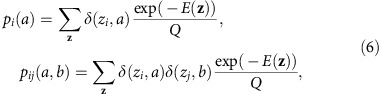


are equal to their counterparts estimated from the MSA, given in equation (2). The problem of determining the *h*_*i*_(*a*)*, J*_*ij*_(*a*,*b*) parameters from the measured correlations is referred to as the inverse Potts problem. Its solution is given by the parameters that maximize the log-likelihood function


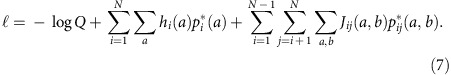


However, no analytical solution exists for systems of nontrivial size, and the likelihood cannot be directly maximized numerically due to the presence of *Q*, which requires summing over a number of terms that grows exponentially with the length of the protein *N*.

To obtain a fast and accurate solution to the inverse Potts problem, we applied an extension of the selective cluster expansion method, described in ref. 27, with computational details in ref. 28. This method was originally developed to solve the inverse Ising problem, a special case of the inverse Potts problem where the number of states at each residue is limited to two. Generalizing the approach to models with an arbitrary number of states at each residue, the algorithm requires maximizing the *L*_2_-regularized likelihood,


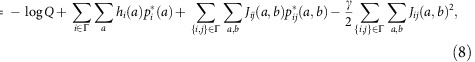


restricted to small subsets *Γ* of the full system, where numerical approaches are feasible. For example, for a two-site subset *Γ*={1,2} we would compute the set of fields *h*_1_(*a*), *h*_2_(*a*) and couplings *J*_12_(*a*,*b*) that maximize the likelihood of the model restricted to just the sites 1 and 2, constrained to reproduce the correlations for those sites *p*_1_*(*a*), *p*_2_*(*a*) and *p*_12_*(*a*,*b*); sites {3, 4, …, *N*} outside of *Γ* are ignored in this calculation.

Using the parameters inferred for many different subsets *Γ*, an approximate solution of the *h*_*i*_(*a*)*, J*_*ij*_(*a*,*b*) for the full system can be constructed^27^,^28^. We follow the procedure described in refs 28 and 13 to infer parameters *h*_*i*_(*a*)*, J*_*ij*_(*a*,*b*) for the Potts model, which accurately recover the measured correlations, without overfitting the model (see also Supplementary Fig. 1). Selection of the optimal regularization strength *γ* was determined by comparing the fit with higher-order statistics of the sequence distribution for models inferred over a range of different *γ*, as detailed in ref. 13. Following a Bayesian interpretation of the *L*_2_-norm regularization term as a Gaussian prior distribution, we naturally expect *γ* to scale as 1/*W*, where W is the number of unique patients from which sequence data from the LANL database were obtained. To ensure that the regularization strength is similar across proteins with comparable sequencing depth, we tested values of the regularization strength ranging from 1/(2 *W*) to 2/*W*. Rather than using the full set of 21 states (20 aa and 1 gap state) at each residue, we used a compressed representation of the states at each residue, as described below.

### Sequence compression

Even with the use of sophisticated algorithms, solving the inverse Potts problem remains a challenging computational task. This task is complicated by the large number of parameters in the model, equal to *N*(*q*-*1*) (*N*(*q*-*1*)+*1*)/*2*, where *N* is the length of the protein sequence and *q* is the number of states, assuming that this number is the same for each residue. Choosing *q*=21 for the 20 possible amino acids plus 1 gap state, we would require more than two million variables to parameterize the Potts model for a protein of length 100, a typical length scale for HIV proteins.

Fortunately, it is not necessary to include all possible amino acids at each residue in the model explicitly to obtain a useful characterization of the sequence distribution. We adaptively adjusted the number of states allowed at each residue based on the frequencies with which different amino acids are observed there in the MSA. Our procedure for choosing the number of states *q*_*i*_ at each residue *i* is as follows. First, we order the amino acids at residue *i* according to how frequently they are observed in the MSA, such that


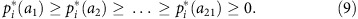


The Shannon entropy of the distribution of amino acids at this residue can be written as


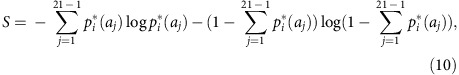


as the *p*_i_*(*a*) must sum to one when summed over all states *a*. Then, we set *q*_*i*_ equal to the smallest integer *q*, such that


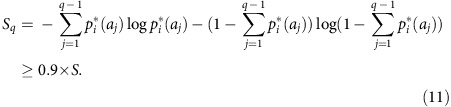


That is, we choose a number of states *q*_*i*_ such that the reduced representation captures at least 90% of the full entropy of the distribution of amino acids at that residue. The *q*_*i*_-1 most frequently observed amino acids at that residue each map to particular Potts states. All the remaining, infrequently observed amino acids map to a single aggregate state.

Our choice of the number of states to model at each residue is adaptive, compressing the amino acid alphabet heavily at residues where little variation is observed, but allowing for a larger number of states when many different amino acids are present at nontrivial frequencies. The particular choice of cutoff given in equation (11) leads to the consideration of multiple states even in conserved proteins such as Gag, while still limiting the number of states sufficiently that the inverse Potts problem remains computationally tractable for the more highly variable proteins studied here, such as Nef and gp41. Successful prediction of higher-order statistics of the sequence distributions suggests that the predictive power of the model is not compromised by our convention for sequence compression (Supplementary Fig. 1).

### Epitope identification

In our study, we included all epitopes identified in ref. 29 that were targeted within 50 days post estimated Fiebig stage I/II, with the exception of epitopes lying in the gp120 subunit of Env, for which we did not obtain a Potts model. This was due to the combination of length and high variability for gp120, which makes the inverse Potts inference problem more difficult. In addition, two Nef epitopes (DEPAAVGVG targeted by CH77 and RIRKTAPTA targeted by CH162) were excluded as a part of these epitopes lie in regions that are insertions relative to the HXB2 reference sequence, and thus not covered by our model. As in ref. 29 we also excluded one epitope where no escape was observed during the course of the study, but sequence data did not extend to at least 200 days from the subject's initial screening visit.

Attempts to identify the optimal epitopes were made in ref. 29, beginning with *ex vivo* IFN-γ ELISPOT assays using overlapping 18 mers matched to the transmitted/founder strain. In 7/71 cases optimal 8–11 mers could not be identified, and hence we used the LANL ELF tool (http://www.hiv.lanl.gov/content/sequence/ELF/epitope_analyzer.html) to search for known HLA-matched epitopes from the LANL CTL database. If no matches were found in the database, we used NetMHC version 3.4 to identify likely epitopes within the reactive 18 mer (ref. 41). We analysed all epitopes that had strong predicted binding affinities (IC_50_≤500 nM). Where possible we used empirically determined HLA-specific cutoffs^42^ rather than the standard threshold of 500 nM. We then averaged the *S*, *ΔE* and *t*_WF_ values across these likely epitopes and used these averages for escape time prediction. The selected epitopes are summarized in Supplementary Table 4. Note that this method for evaluating epitopes that could not be directly identified differs from that used in ref. 29.

In total, the distribution of the 71 epitopes we considered among HIV proteins is as follows. We analysed 24 epitopes from Gag: 4 epitopes from p17, 16 from p24, 3 from p7 and 1 from p6. From Pol, we analysed 5 epitopes: 1 from protease, 3 from reverse transcriptase and 1 from integrase. Our study includes 7 epitopes from the regulatory proteins: 3 from Tat and 4 from Rev. We analysed 12 epitopes from the gp41 subunit protein of Env and 23 epitopes from the accessory proteins: 4 from Vif, 1 from Vpr and 18 from Nef. See Supplementary Data 1 for a list of epitopes and their properties.

### Estimation of escape times from clinical data

Limited numbers of sample sequences and long delays between sampling times make reliable inference of escape times difficult. To combat this issue, we used a mathematical method developed to infer the kinetics of viral escape from T-cell pressure^33^ to provide a robust estimation of time to escape. Briefly, the growth in the fraction of escape mutants in the virus population over time can be approximated by a logistic equation


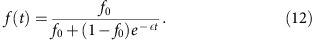


Here, *f*(*t*) is the fraction of escape mutants in the population over time, *f*_0_ is the initial fraction of escape mutants, and *ɛ* is a parameter that expresses the rate of growth of the escape mutants relative to the rest of the virus population. The parameters *f*_0_ and *ɛ* appearing in equation (12) can be estimated from time series sequence data: given a collection of sequences ***n***={*n*_1_, *n*_2_, …, *n*_T_} collected at times ***t***={*t*_1_, *t*_2_, …, *t*_*T*_}, the likelihood of observing a number of escape mutants ***k***={*k*_1_, *k*_2_, …, *k*_T_} assuming that the true fraction of escape mutants in the population obeys equation (12) is^33^


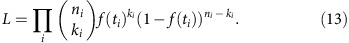


For each epitope we thus obtained maximum likelihood estimates of *f*_0_ and *ɛ*, and then used these parameters in equation (12) to solve for the time at which the fraction of escape mutants in the population was equal to 50%,


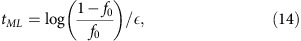


which we refer to as the maximum likelihood escape time. The threshold of 50% escape mutants in the population was chosen to reflect previously used definitions of escape time^29^. If no sequences were available at the precise time that the T-cell response was first detected, we used the most recently collected sequences for the first time point. We included a lower bound of 1 day on escape times, so that escapes inferred to occur in ≤1 day were rounded up to one. The overall correlation between the maximum likelihood escape time and those computed in previous work^29^ is strong (Pearson's *r*=0.92, *P*=3.8 × 10^−30^, *n*=71), but the maximum likelihood approach tends to yield shorter escape times in cases where escape is rapid. This method was used to estimate the escape time for both conventional escapes (through mutations within an epitope) and escapes occurring through putative antigen-processing mutations.

### Prediction of fitness costs of escape mutations

The difference in energy between sequences can be used to quantify their expected difference in fitness. This assertion is supported by *in vitro* tests of viral replicative capacity for multiple closely related HIV strains, which found a strong correlation between differences in energy and replicative capacity^10^,^13^. We can thus compute the energy difference between a sequence and potential escape mutants to quantify the expected fitness barrier to mutational escape in a targeted epitope.

For each targeted epitope, we began with the transmitted/founder (T/F) sequence **z** for the viral protein in which that epitope is located. In case the T/F sequence was not available, we used the most common sequence in the virus population at the earliest time point when sequencing data were available. We then generate the set of all sequences {**z**′} that differ from **z** by a single non-synonymous mutation in the targeted T-cell epitope, and compute the average difference in energy between this set of sequences and **z**:


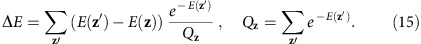


This Boltzmann-like average emphasizes the contribution of the escape mutation with the lowest fitness cost. Focusing only on sequences {**z**′} that differ by a single nucleotide mutation from **z** allows us to estimate the fitness cost of the shortest mutational path to escape. More involved escape trajectories are effectively taken into account when we simulate the evolution of the virus population, as described below.

### Evolutionary simulations

To simulate the evolution of virus populations *in vivo*, we coupled the inferred Potts model to a Wright–Fisher-like evolutionary model. We assume a fixed population size of *N*=10^4^ viruses in the population, in line with estimates of the effective population size of HIV for intra-host evolution^43^. In each run of the simulation, the fraction of each sequence in the starting virus population is taken to be the same as in the set of viral sequences collected at the time point that the T-cell response was first detected. If there were no sequence data available from the same time that the T-cell response was detected, we used the most recently collected sequences before that time to set the fraction of each sequence in the virus population.

The starting population of sequences then evolves in discrete time steps, with rounds of selection, replication and mutation. In the selection step, each sequence **z** survives with probability


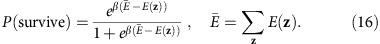


This form of the survival probability smoothly interpolates between *P*=0, for sequences that are much less fit (that is, much higher energy) than the rest of the population, and *P*=1, for sequences that are much fitter than the population average. Using experimental measurements of viral replicative capacities and sequence energies for a set of Gag mutants^13^, independent from the current study, we estimated β≈0.07 (Supplementary Fig. 4). Choosing other values of β≤0.1 also leads to similar results. After each selection step, the population size is restored to *N* by random resampling with replacement from the survivors. Following the replication step, each sequence mutates with rate *μ*=3 × 10^−5^ per base, in line with known HIV mutation rates^44^. Sequences can then recombine with rate *ρ*=1 × 10^−5^ per base, following recent estimates of HIV recombination rates^45^,^46^. To account for the effect on viral replication of the killing of infected cells by T cells specific for the targeted epitope, sequences without non-synonymous mutations in the targeted epitope had their energies increased (that is, fitness decreased) by *b*=10, a value chosen to be larger than the largest *ΔE* (average cost of escape) so that escape confers a selective advantage for all epitopes. To quantify the ease of escape at each epitope we computed the number of generations to escape (≥50% of escape mutants in the population), averaged over 10^3^ simulations. The predicted order of escape is not sensitive to the precise values of β and *b*, provided that the latter is larger than the largest *ΔE*. Choosing *b*=9 or *b*=11, for example, leads to virtually identical values for the correlation between the escape generation *t*_WF_ and the escape time for all epitopes (Pearson's *r*=0.79 for *b*=9, and *r*=0.81 for *b*=11, *n*=71), but larger values of *b* lead to shorter average escape times <*t*_WF_> across all epitopes (<*t*_WF_>=32.5, 28.8 and 26.0 for *b*=9, 10 and 11, respectively).

We note that, if the fitness penalty *b* applied to viruses lacking escape mutations is small enough so that all mutations within the targeted epitope are deleterious even including the fitness benefit of escape, then clearly escape would be observed only after extremely long periods of time. Indeed, we expect that in some real cases the fitness cost of escape mutations in an epitope can be high enough that no selective advantage is gained through escape, and thus escape mutants never come to dominate the population. In the present work, our goal is to predict the relative ease of generating escape mutations in each targeted epitope; thus we have chosen *b* large enough that escape is preferred in all the epitopes we considered. The average escape generations computed through the simulation described above should therefore be interpreted as relative rates for the evolution of escape mutants for each epitope.

As an example, escape at the Gag epitope ASRELERF_37–44_ targeted by patient CH77, which has the highest *ΔE* (=6.6) of all epitopes we considered, is never observed. With *b*=10, the mean escape generation in the Wright–Fisher simulation is 52.9, also the largest among all epitopes. As *b* approaches *ΔE* the value of *t*_WF_ begins to increase sharply as escape no longer confers a large selective advantage (*t*_WF_=69.6 and 122.4 for *b*=9 and 8, respectively). Selecting *b*≥9 avoids this threshold effect for epitopes with the highest *ΔE*.

### Incorporating the effects of immunodominance in evolutionary simulations

As shown above and in ref. 29, the initial vertical immunodominance (%*M*) of each CD8^+^ T-cell response influences the rate of escape. More vigorous immune responses increase the selective pressure for escape, and thus escape occurs more rapidly at epitopes where the vertical immunodominance is higher. We can incorporate this factor into the Wright–Fisher simulation by increasing the fitness penalty *b* for viruses without escape mutations in proportion with the strength of the immune response directed towards each epitope: *b*=(1−%*M*) *b*_*min*_+%*M b*_*max*_. To avoid extremely long escape times for epitopes with the highest *ΔE*, we took *b*_*min*_=9 and *b*_*max*_=2 *b*_*min*_. We then computed the average escape time *t*_WF_ for the set of epitopes for which vertical immunodominance measurements are available, incorporating this immunodominance-dependent *b*. For these epitopes, incorporating the effects of immunodominance does not result in significant changes in the Pearson correlation with the inferred escape times (*r*=0.81, *P*=3 × 10^−13^ with immunodominance-dependent *b* versus *r*=0.80, *P*=1 × 10^−12^ without, *n*=53; includes 4 escapes at the time the T-cell response was first detected), but the rank correlation is substantially improved due to better ordering of epitopes with intermediate predicted escape times (*r*=0.73, *P*=4 × 10^−10^ with immunodominance-dependent *b* versus *r*=0.53, *P*=4 × 10^−5^ without). As before, provided that *b*_*min*_ is large enough to avoid threshold effects for epitopes with the largest values of *ΔE*, our results are not sensitive to the precise values of the parameters (for example, with *b*_*min*_=9 we find Spearman's *r*=0.734, 0.739 for *b*_*max*_=2 *b*_*min*_−1, 2 *b*_*min*_+1, respectively).

### Effects of escape mutants in the initial population on escape predictions

For 11 epitopes, the sample of the virus population at the time that the T-cell response towards that epitope was first detected already contains one or more escape mutants. These cases represent instances where either testing of T-cell responses was performed too late to detect the response before escape began, or where assays performed at earlier times had insufficient sensitivity to detect T-cell responses before escape occurred. This uncertainty in the exact timing of the T-cell response is large in proportion to the estimated escape time for epitopes where escape occurs rapidly. Because we are unable to infer the precise time that the T-cell response was initiated (and the composition of the virus population at exactly that time), we have used the available sequence data at the time the T-cell response was first detected as the basis of our evolutionary simulations.

One can also consider the effects of excluding these epitopes from analysis. This results in reduced correlation between the inferred escape time and both the escape time in simulated evolution and the fitness cost of escape mutations (see Supplementary Table 5). This is because the fitness cost of escape at epitopes where escape mutants are observed at the time when the T-cell response is first detected is lower than that for other epitopes (*t*=−2.27, *P*=0.035, *n*=71, two sample *t*-test). To a lesser extent, these epitopes also tend to be more immunodominantly targeted (*t*=1.62, *P*=0.133, *n*=53 epitopes with available immunodominance information, two sample *t*-test). These epitopes thus represent a select sample where the fitness cost of escape is unusually low and where rapid escape is successfully predicted, arguing against their exclusion. Alternatively, reverting observed escape mutations in the sequence data and the time the T-cell response was first detected and using these reverted sequences as a starting point for evolutionary simulations also recover rapid escape times for these epitopes, but overall correlation is lowered in this case because of the inaccurate estimation of the true time that the T-cell response was initiated for these epitopes (see Supplementary Table 5).

### Effects of immunodominance on escape and comparison with other predictors

We used a CPH model to quantify the influence of fitness, epitope entropy and relative immunodominance on rates of escape. Here we restricted our attention to the set of *n*=53 epitopes for which relative immunodominance data were available. Cases where escape either was not observed (*n*=6) or occurred through putative antigen-processing (AgP) mutation outside the epitope (*n*=3) were treated as censored events. Incorporating vertical immunodominance in a multivariate model considerably improves the model fit for epitope entropy and the fitness cost of escape (pseudo-*R*^2^=0.37 and 0.42 excluding 4 epitopes with escapes at the time the T-cell response was first detected, Table 1), with a smaller improvement for time to escape in simulated evolution (pseudo-*R*^2^=0.56). We repeated the same analysis for patient-stratified CPH models, which include variable escape rates for each patient. Although the predictive power is weaker in this case, the overall results here are similar to those described above (Supplementary Table 3).

### Data availability

Summarized data on targeted epitopes are included in Supplementary Data 1. All other data supporting the findings of this study are available from the corresponding authors upon request.

## Additional information

**How to cite this article:** Barton, J. P. *et al*. Relative rate and location of intra-host HIV evolution to evade cellular immunity are predictable. *Nat. Commun.* 7:11660 doi: 10.1038/ncomms11660 (2016).

## Supplementary Material

Supplementary Information Supplementary Figures 1-7, Supplementary Tables 1-5, Supplementary References

Supplementary Dataset 1 List of targeted epitopes organized by patient, including information about the estimated time to escape, epitope entropy (S), estimated fitness cost of escape mutations (ΔE), time to escape in simulated evolution (tWF), and vertical immunodominance (%M, if available). Users of this data in a subsequent paper should also cite the primary experimental source (Liu, M. K. P. et al., J Clin Invest 123, 380-393 (2013)).

## Figures and Tables

**Table 1:** In cases where identical epitopes are targeted by multiple individuals, escape occurs more rapidly when the fitness cost of escape is lower.

Epitope	Patient	HLA restriction	Fitness cost Δ*E*	Escape time (days)
TPQDLNTML (TL9)	CH185	B*81:01	4.3	122
	CH159	B*81:01	6.1	>1,103*
TSTLQEQVAW (TW10)	CAP239	B*58:01	−1.4	1
	CH198	B*57:03	0.1	220
WHLGHGVSI (WI9)	CAP210	B*15:10	2.8	127
	CAP45	B*15:10	4.7	408†
EEVGFPVRPQV (EV11)	CH164	B*45:01	2.6	31
	CAP45	B*45:01	4.0	43

^*^No escape observed (final sequencing time).

^†^Antigen-processing escape.

**Table 2:** Cox proportional hazards models quantify contributions to escape rate.

Predictors	Coefficient	*P* value	Pseudo-*R*^2^
*Univariate models (*n*=53 epitopes, maximum possible pseudo*-R^*2*^*=0.99)*			
log_10_(*S*)	0.87	0.08	0.06
*ΔE*	−0.14	0.02	0.10
*t*_WF_	−0.14	5.8 × 10^−8^	0.51
*t*_WF_^%M^	−0.17	1.5 × 10^−9^	0.63
log_10_(%*M*)	1.53	7.8 × 10^−5^	0.29
			
*Multivariate models (*n*=53, maximum possible pseudo*-R^*2*^*=0.99)*			
log_10_(*S*)+ log_10_(%*M*)	1.111.60	0.079.3 × 10^−5^	0.33
*ΔE*+ log_10_(%*M*)	−0.171.66	4.4 × 10^−3^3.6 × 10^−5^	0.39
*t*_WF_+ log_10_(%*M*)	−0.141.55	1.3 × 10^−7^1.7 × 10^−4^	0.64
*t*_WF_^%M^+ log_10_(%*M*)	−0.160.13	1.7 × 10^−7^0.76	0.64
			
*Univariate models, excluding escapes at the time the T-cell response was first detected (*n*=49, maximum possible pseudo*-R^*2*^*=0.99)*			
log_10_(*S*)	0.81	0.11	0.05
*ΔE*	−0.14	0.02	0.10
*t*_WF_	−0.12	8.9 × 10^−6^	0.37
*t*_WF_^%M^	−0.15	1.1 × 10^−7^	0.53
log_10_(%*M*)	1.68	5.1 × 10^−5^	0.33
			
*Multivariate models, excluding escapes at the time the T-cell response was first detected (*n*=49, maximum possible pseudo*-R^*2*^*=0.99)*			
log_10_(*S*)+ log_10_(%*M*)	1.061.77	0.106.2 × 10^−5^	0.37
*ΔE*+ log_10_(%*M*)	−0.181.83	5.0 × 10^−3^2.3 × 10^−5^	0.42
*t*_WF_+ log_10_(%*M*)	−0.121.65	2.0 × 10^−5^1.1 × 10^−4^	0.56
*t*_WF_^%M^+ log_10_(%*M*)	−0.140.37	4.8 × 10^−5^0.43	0.54

Contributions of vertical immunodominance (%*M*) and purely fitness-related measures (*S*, *ΔE* and *t*_WF_) are mostly independent.

## References

[b1] Phillips R. E. . Human immunodeficiency virus genetic variation that can escape cytotoxic T cell recognition. Nature 354, 453–459 (1991). 1721107 10.1038/354453a0

[b2] McMichael A. J., Borrow P., Tomaras G. D., Goonetilleke N. & Haynes B. F. The immune response during acute HIV-1 infection: clues for vaccine development. Nat. Rev. Immunol. 10, 11–23 (2009). 20010788 10.1038/nri2674 PMC3119211

[b3] Feeney M. E. . Immune escape precedes breakthrough human immunodeficiency virus type 1 Viremia and broadening of the cytotoxic T-lymphocyte response in an HLA-B27-positive long-term-nonprogressing child. J. Virol. 78, 8927–8930 (2004). 15280502 10.1128/JVI.78.16.8927-8930.2004 PMC479057

[b4] Allen T. M. . Selective escape from CD8+ T-cell responses represents a major driving force of human immunodeficiency virus type 1 (HIV-1) sequence diversity and reveals constraints on HIV-1 evolution. J. Virol. 79, 13239–13249 (2005). 16227247 10.1128/JVI.79.21.13239-13249.2005 PMC1262562

[b5] Draenert R. . Constraints on HIV-1 evolution and immunodominance revealed in monozygotic adult twins infected with the same virus. J. Exp. Med. 203, 529–539 (2006). 16533886 10.1084/jem.20052116 PMC2118231

[b6] Dahirel V. . Coordinate linkage of HIV evolution reveals regions of immunological vulnerability. Proc. Natl Acad. Sci. USA 108, 11530–11535 (2011). 21690407 10.1073/pnas.1105315108 PMC3136285

[b7] Martinez-Picado J. . Fitness cost of escape mutations in p24 Gag in association with control of human immunodeficiency virus type 1. J. Virol. 80, 3617–3623 (2006). 16537629 10.1128/JVI.80.7.3617-3623.2006 PMC1440414

[b8] Brockman M. A. . Escape and compensation from early HLA-B57-mediated cytotoxic T-lymphocyte pressure on human immunodeficiency virus type 1 Gag alter capsid interactions with cyclophilin A. J. Virol. 81, 12608–12618 (2007). 17728232 10.1128/JVI.01369-07 PMC2169025

[b9] Hinkley T. . A systems analysis of mutational effects in HIV-1 protease and reverse transcriptase. Nat. Genet. 43, 487–489 (2011). 21441930 10.1038/ng.795

[b10] Ferguson A. L. . Translating HIV sequences into quantitative fitness landscapes predicts viral vulnerabilities for rational immunogen design. Immunity 38, 606–617 (2013). 23521886 10.1016/j.immuni.2012.11.022 PMC3728823

[b11] Pandit A. & De Boer R. J. Reliable reconstruction of HIV-1 whole genome haplotypes reveals clonal interference and genetic hitchhiking among immune escape variants. Retrovirology 11, 56 (2014). 24996694 10.1186/1742-4690-11-56 PMC4227095

[b12] Goulder P. J. R. & Walker B. D. HIV and HLA class I: an evolving relationship. Immunity 37, 426–440 (2012). 22999948 10.1016/j.immuni.2012.09.005 PMC3966573

[b13] Mann J. K. . The fitness landscape of HIV-1 Gag: advanced modeling approaches and validation of model predictions by in vitro testing. PLoS. Comput. Biol. 10, e1003776 (2014). 25102049 10.1371/journal.pcbi.1003776 PMC4125067

[b14] Jaynes E. T. On the rationale of maximum-entropy methods. P. IEEE 70, 939–952 (1982).

[b15] Mora T. & Bialek W. Are biological systems poised at criticality? J. Stat. Phys. 144, 268–302 (2011).

[b16] Mora T., Walczak A. M., Bialek W. & Callan C. G. Maximum entropy models for antibody diversity. Proc. Natl Acad. Sci. USA 107, 5405 (2010). 20212159 10.1073/pnas.1001705107 PMC2851784

[b17] Weigt M., White R. A., Szurmant H., Hoch J. A. & Hwa T. Identification of direct residue contacts in protein-protein interaction by message passing. Proc. Natl Acad. Sci. USA 106, 67–72 (2009). 19116270 10.1073/pnas.0805923106 PMC2629192

[b18] Berg J., Willmann S. & Lässig M. Adaptive evolution of transcription factor binding sites. BMC Evol. Biol. 4, 42 (2004). 15511291 10.1186/1471-2148-4-42 PMC535555

[b19] Sella G. & Hirsh A. E. The application of statistical physics to evolutionary biology. Proc. Natl Acad. Sci. USA 102, 9541–9546 (2005). 15980155 10.1073/pnas.0501865102 PMC1172247

[b20] Goldrath A. W. & Bevan M. J. Selecting and maintaining a diverse T-cell repertoire. Nature 402, 255–262 (1999). 10580495 10.1038/46218

[b21] Friedrich T. C. . Reversion of CTL escape-variant immunodeficiency viruses *in vivo*. Nat. Med. 10, 275–281 (2004). 14966520 10.1038/nm998

[b22] Korber B. . Evolutionary and immunological implications of contemporary HIV-1 variation. Brit. Med. Bull. 58, 19–42 (2001). 11714622 10.1093/bmb/58.1.19

[b23] Łuksza M. & Lässig M. A predictive fitness model for influenza. Nature 507, 57–61 (2014). 24572367 10.1038/nature13087

[b24] Barton J. P., Kardar M. & Chakraborty A. K. Scaling laws describe memories of host–pathogen riposte in the HIV population. Proc. Natl Acad. Sci. USA 112, 1965–1970 (2015). 25646424 10.1073/pnas.1415386112 PMC4343148

[b25] Shekhar K. . Spin models inferred from patient-derived viral sequence data faithfully describe HIV fitness landscapes. Phys. Rev. E 88, 062705 (2013). 10.1103/PhysRevE.88.062705 PMC5260469 24483484

[b26] Zanini F. . Population genomics of intrapatient HIV-1 evolution. eLife 4, 13239 (2015). 10.7554/eLife.11282 PMC4718817 26652000

[b27] Cocco S. & Monasson R. Adaptive cluster expansion for inferring Boltzmann machines with noisy data. Phys. Rev. Lett. 106, 090601 (2011). 21405611 10.1103/PhysRevLett.106.090601

[b28] Barton J. & Cocco S. Ising models for neural activity inferred via selective cluster expansion: structural and coding properties. J. Stat. Mech. 2013, P03002 (2013).

[b29] Liu M. K. P. . Vertical T cell immunodominance and epitope entropy determine HIV-1 escape. J. Clin. Invest. 123, 380–393 (2013). 23221345 10.1172/JCI65330 PMC3533301

[b30] Goonetilleke N. . The first T cell response to transmitted/founder virus contributes to the control of acute viremia in HIV-1 infection. J. Exp. Med. 206, 1253–1272 (2009). 19487423 10.1084/jem.20090365 PMC2715063

[b31] Streeck H. . Human immunodeficiency virus type 1-specific CD8+ T-cell responses during primary infection are major determinants of the viral set point and loss of CD4+ T cells. J. Virol. 83, 7641–7648 (2009). 19458000 10.1128/JVI.00182-09 PMC2708622

[b32] Fiebig E. W. . Dynamics of HIV viremia and antibody seroconversion in plasma donors: implications for diagnosis and staging of primary HIV infection. AIDS. 17, 1871–1879 (2003). 12960819 10.1097/00002030-200309050-00005

[b33] Ganusov V. V., Neher R. A. & Perelson A. S. Mathematical modeling of escape of HIV from cytotoxic T lymphocyte responses. J. Stat. Mech. 2013, P01010 (2013). 24660019 10.1088/1742-5468/2013/01/P01010 PMC3961578

[b34] Carlson J. M. . Selection bias at the heterosexual HIV-1 transmission bottleneck. Science 345, 1254031–1254031 (2014). 25013080 10.1126/science.1254031 PMC4289910

[b35] Deng K. . Broad CTL response is required to clear latent HIV-1 due to dominance of escape mutations. Nature 517, 381–385 (2015). 25561180 10.1038/nature14053 PMC4406054

[b36] Lee J. K. . T cell cross-reactivity and conformational changes during TCR engagement. J. Exp. Med. 200, 1455–1466 (2004). 15583017 10.1084/jem.20041251 PMC2211951

[b37] Huseby E. S. . How the T cell repertoire becomes peptide and MHC specific. Cell 122, 247–260 (2005). 16051149 10.1016/j.cell.2005.05.013

[b38] Huseby E. S., Crawford F., White J., Marrack P. & Kappler J. W. Interface-disrupting amino acids establish specificity between T cell receptors and complexes of major histocompatibility complex and peptide. Nat. Immunol. 7, 1191–1199 (2006). 17041605 10.1038/ni1401

[b39] Košmrlj A., Jha A. K., Huseby E. S., Kardar M. & Chakraborty A. K. How the thymus designs antigen-specific and self-tolerant T cell receptor sequences. Proc. Natl Acad. Sci. USA 105, 16671–16676 (2008). 18946038 10.1073/pnas.0808081105 PMC2575478

[b40] Ritchie A. J. . Recombination-mediated escape from primary CD8+ T cells in acute HIV-1 infection. Retrovirology 11, 1–10 (2014). 25212771 10.1186/s12977-014-0069-9 PMC4180588

[b41] Lundegaard C. . NetMHC-3.0: accurate web accessible predictions of human, mouse and monkey MHC class I affinities for peptides of length 8-11. Nucleic Acids Res. 36, W509–W512 (2008). 18463140 10.1093/nar/gkn202 PMC2447772

[b42] Paul S. . HLA class I alleles are associated with peptide-binding repertoires of different size, affinity, and immunogenicity. J. Immunol. 191, 5831–5839 (2013). 24190657 10.4049/jimmunol.1302101 PMC3872965

[b43] Achaz G. . A robust measure of HIV-1 population turnover within chronically infected individuals. Mol. Biol. Evol. 21, 1902–1912 (2004). 15215321 10.1093/molbev/msh196

[b44] Sanjuan R., Nebot M. R., Chirico N., Mansky L. M. & Belshaw R. Viral mutation rates. J. Virol. 84, 9733–9748 (2010). 20660197 10.1128/JVI.00694-10 PMC2937809

[b45] Neher R. A. & Leitner T. Recombination rate and selection strength in HIV intra-patient evolution. PLoS Comput. Biol. 6, e1000660 (2010). 20126527 10.1371/journal.pcbi.1000660 PMC2813257

[b46] Batorsky R. . Estimate of effective recombination rate and average selection coefficient for HIV in chronic infection. Proc. Natl Acad. Sci. USA 108, 5661–5666 (2011). 21436045 10.1073/pnas.1102036108 PMC3078368

