# Supplementary material for: Relative rate and location of intra-host HIV evolution to evade cellular immunity are predictable
Source: Nat Commun. 2016 May 23;7:11660. doi: 10.1038/ncomms11660 (PMC4879252; doi:10.1038/ncomms11660)
Supplement: Supplementary Information — Supplementary Figures 1-7, Supplementary Tables 1-5, Supplementary References [file ncomms11660-s1.pdf]

Supplementary figures

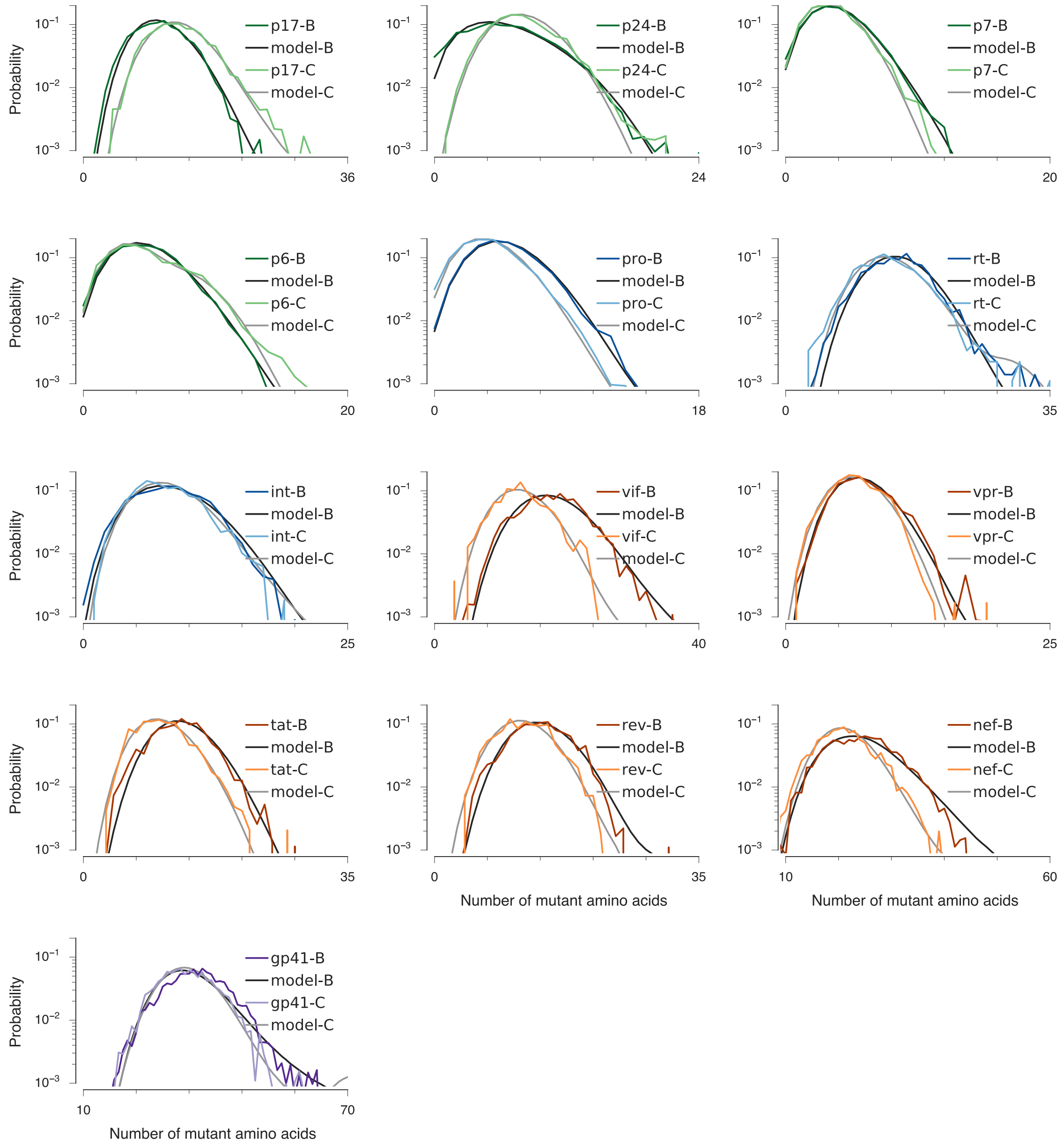

Supplementary Figure 1 | The Potts model accurately reproduces higher order statistics of the HIV sequence distribution, which are not directly constrained by the inverse Potts inference procedure. The probability of observing a sequence with a certain number of mutant amino acids relative to the consensus sequence is not directly constrained by the inverse Potts inference problem. However, inferred Potts models accurately reproduce this probability distribution, demonstrating that they effectively capture important constraints on HIV sequences beyond pairwise mutational probabilities.

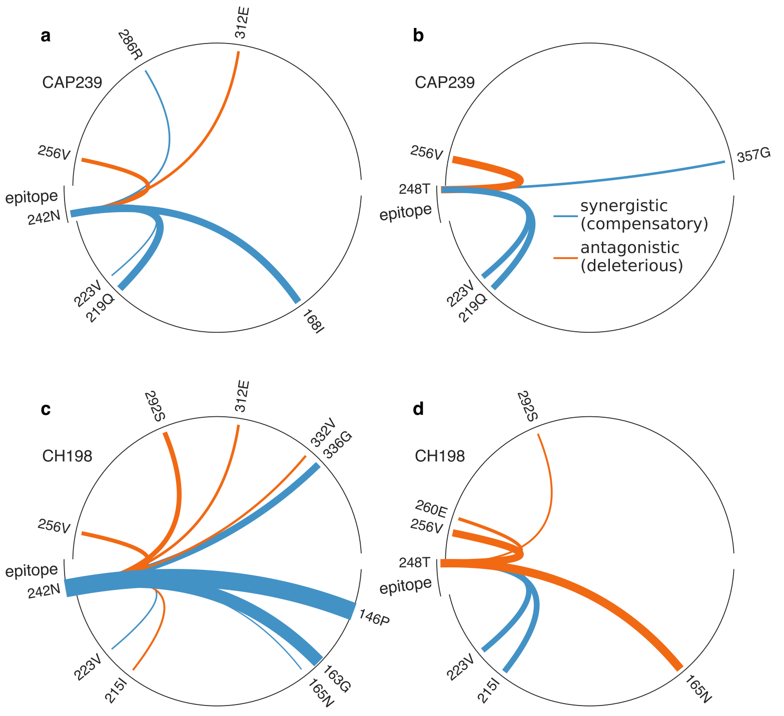

Supplementary Figure 2 | Different sequence backgrounds lead to different patterns of escape in the Gag TW10 epitope. Strong interactions between the Gag TW10 epitope escape mutations 242N (**a**) and 248T (**b**) and specific residues in the sequence background in patient CAP239 lower the fitness cost of these two mutations. All strong interactions ($|J|>0.1$, see equation (1) in the main text) between these escape mutations and the p24 protein sequence background are shown, with the width of the link proportional to the magnitude of the coupling. 223V and 219Q are known compensatory mutations. Similarly, 146P has been positively associated with variation in the TW10 epitope[1], and 256V is known to strongly suppress TW10 variation[2]. In patient CAP239, escape occurs through mutations 242N and 248T. Compensatory residues in the sequence background in patient CH198 lower the fitness cost of the 242N escape mutation (**c**), but other escape mutations such as 248T are suppressed (**d**). In patient CH198, escape occurs only through the 242N mutation.

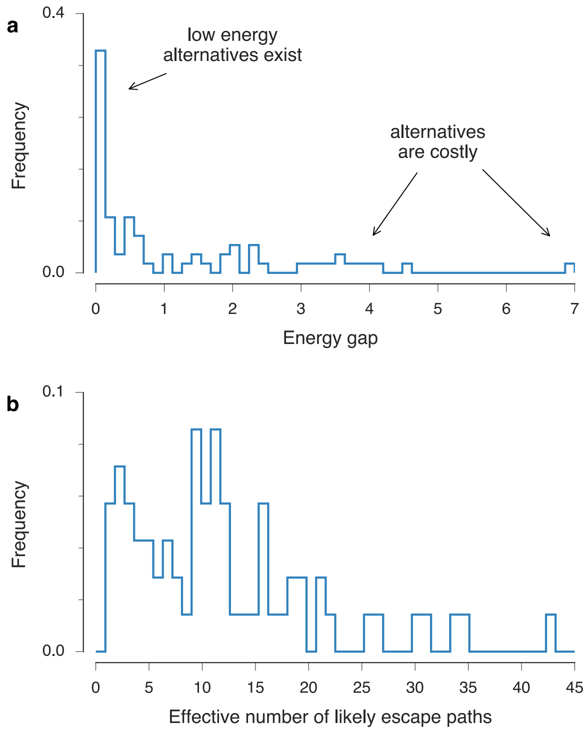

Supplementary Figure 3 | Exploring the potential contributions of multiple escape pathways. (**a**) Difference in energy (gap) between the predicted fittest and second fittest potential escape mutants for each epitope. When the gap is large, this indicates that alternative escape mutations may come at a much larger fitness cost to the virus, compared to the easiest escape path. In contrast, a low value for the gap indicates that multiple alternative escape routes with similar fitness costs exist. Typically, multiple potential escape mutations are available that have comparable fitness costs, but in some cases the fitness cost of escape increases sharply for suboptimal escape paths. (**b**) Logarithm of the entropy of the sequence distribution (see equation (1) in the main text) restricted to the set of escape mutant sequences for each epitope only, which can be interpreted as an effective number of likely escape paths.

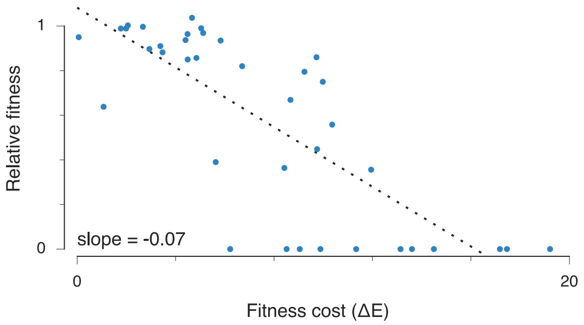

Supplementary Figure 4 | Empirical correlation between viral replicative capacity and energy. Using experimental measurements of viral replicative capacity[3] taken from a study unrelated to this work, along with corresponding energy measurements for these viral sequences, we can derive an empirical relationship for variation in viral fitness as a function of energy.

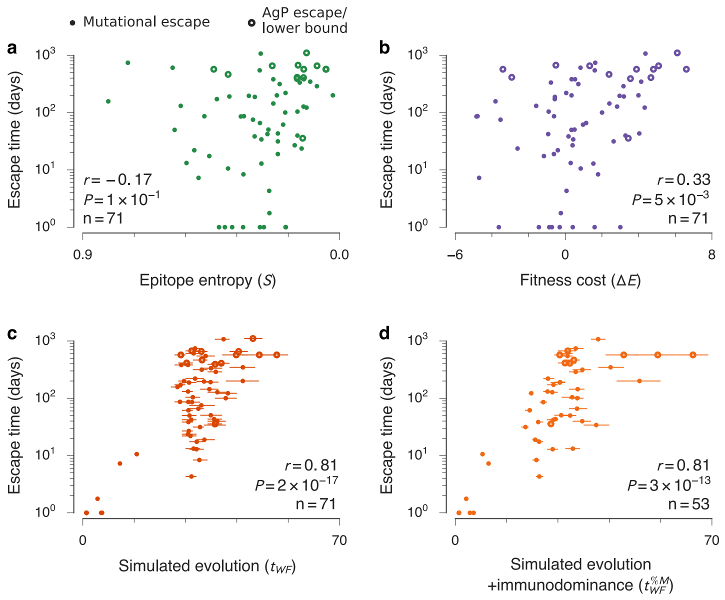

Supplementary Figure 5 | Correlation between escape time and fitness-based measures can improve when epitopes where escape is observed at the time the T cell response was first detected are included. (**a-d**) analogous to Fig. 2 in the main text, including epitopes where ≥50% of the virus population consists of escape mutants at the time the T cell response was first detected. Total of n=71 epitopes, including 3 epitopes where escape occurs through putative antigen processing (AgP) mutation outside the epitope, and 10 epitopes where no escape is observed. Vertical immunodominance measurements are available for a subset (n=53) of these epitopes. Error bars show first/third quartiles for time to escape in the Wright-Fisher simulations, computed from the statistics of 10^3 simulation runs.

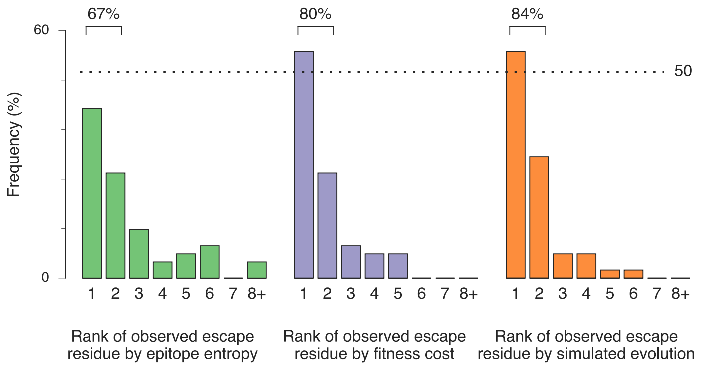

Supplementary Figure 6 | Fitness-based methods accurately predict the residues at which escape mutations occur. In the great majority of epitopes, the most common residue where escape mutations are observed in patients during the entire time course of evolution corresponds to one of the two top residues where escape mutations are predicted to incur the lowest fitness costs (41/51=80% of epitopes where escape is observed) or where mutations are most frequently observed in simulated evolution (43/51=84%). Less frequently, the residue where escape mutations are observed most often has one of the top two highest Shannon entropies (34/51=67%). Epitopes where escape was observed at the time the T cell response was detected are excluded (*n*=6), as is one epitope without detailed escape sequence data.

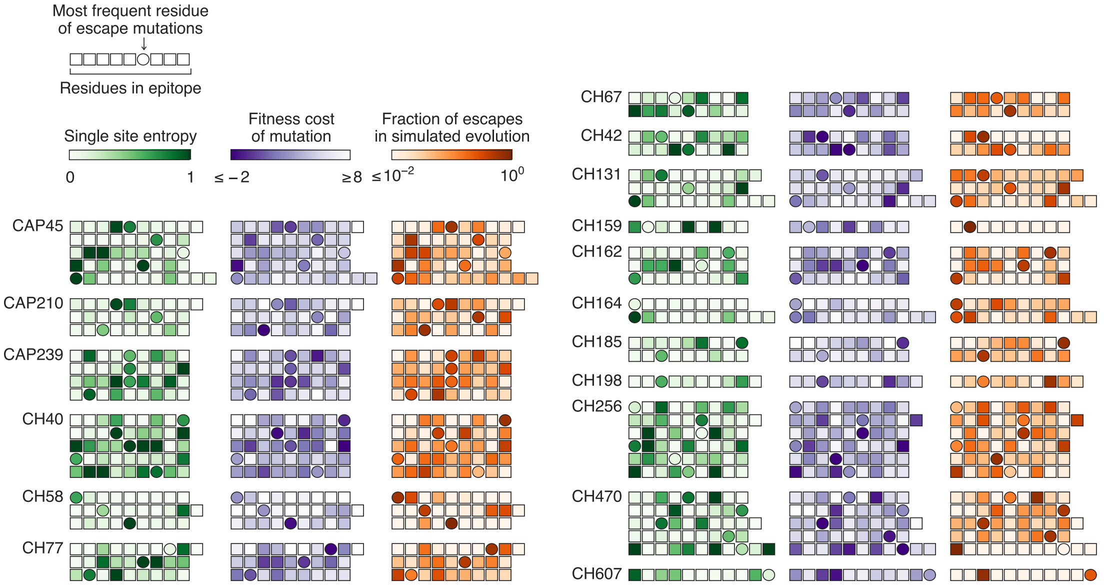

Supplementary Figure 7 | Predicting the residues of escape mutations in individual epitopes. Here we show the single site entropy, fitness cost of mutation, and frequency of escape mutations in simulated evolution at each residue for all epitopes where nonsynonymous mutations were observed in the epitope (*n*=51). Each epitope is represented by a row of residues, with the residue where escape mutations were most frequently observed in the clinical data denoted by a circle. Predictions for the same epitope based on epitope entropy, fitness cost, and simulated evolution are placed side by side in each row. Darker colors indicate residues where escape mutations are predicted to be more likely. Predictions are correct when the circle in each row is more darkly shaded than the boxes in the same row. Epitopes where escape was observed at the time the T cell response was detected are excluded (*n*=6), as is one epitope without detailed escape sequence data.

Supplementary tables

Supplementary Table 1 | Sequence data used to train the Potts model

Protein	Number of sequences (clade B)	Number of individuals (clade B)	Number of sequences (clade C)	Number of individuals (clade C)
p17	8787	4695	6076	2374
p24	8921	4882	9105	2364
p7	7801	3838	8361	2013
p6	8189	4064	5561	2346
pro	14786	10263	5387	4315
RT	2260	1434	1526	894
int	4889	2785	1993	1118
vif	6450	1851	3483	544
vpr	5670	1603	3524	597
tat	3315	875	2478	485
rev	3340	904	2561	550
vpu	4865	1494	3208	713
gp41	17366	2063	11903	1397
nef	8734	2586	4098	1197

Our analysis employs HIV sequence data broadly sampled from thousands of individuals infected by both clade B and clade C viruses, far beyond the cohort of 17 individuals considered here, in order to obtain a more accurate estimate of the distribution of HIV sequences at the population level. Here we report the total number of sequences (and the number of unique individuals from which they were obtained) used to train the Potts models for each protein/clade. All sequences were downloaded from the Los Alamos National Laboratory HIV sequence database (www.hiv.lanl.gov). In order to reduce the influence of selection for drug resistance, only sequences from drug-naïve individuals were used for protease and reverse transcriptase.

Supplementary Table 2 | Rank correlations between predictors and escape time for the set of epitopes with known immunodominance

	**Predictor**	**Spearman correlation**	***P* value**
Including escapes at the time the T cell response was first detected (*n*=53 epitopes)	S	-0.22	0.12
	ΔE	0.38	5.3×10^{-3}
	t_{WF}	0.53	4.2×10^{-5}
	$t_{\mathrm{WF}}^{\%\mathrm{M}}$	0.73	4.4×10^{-10}
	$\%M$	-0.53	4.0×10^{-5}
Excluding escapes at the time the T cell response was first detected (*n*=49)	S	-0.20	0.16
	ΔE	0.37	9.9×10^{-3}
	t_{WF}	0.41	3.6×10^{-3}
	$t_{\mathrm{WF}}^{\%\mathrm{M}}$	0.66	2.1×10^{-7}
	$\%M$	-0.60	4.3×10^{-6}

Supplementary Table 3 | Patient-stratified Cox proportional hazards models

	Predictors	Coefficient	*P* value	Pseudo-R^2
Univariate models	$\log_{10}(S)$	1.52	0.09	0.06
(n=53 epitopes, maximum	ΔE	-0.22	0.04	0.08
possible pseudo-R^2=0.72)	t_{WF}	-0.13	2.4×10^{-3}	0.31
	$t_{WF}^{\%M}$	-0.16	1.0×10^{-3}	0.40
	$\log_{10}(\%M)$	1.69	9.3×10^{-3}	0.14
Multivariate models	$\log_{10}(S)$ +	2.26	0.05	0.22
(n=53, maximum possible	$\log_{10}(\%M)$	1.97	7.1×10^{-3}	
pseudo-R^2=0.72)	ΔE +	-0.34	0.02	0.25
	$\log_{10}(\%M)$	2.02	2.8×10^{-3}	
	t_{WF} +	-0.16	4.9×10^{-3}	0.44
	$\log_{10}(\%M)$	2.25	7.1×10^{-3}	
	$t_{WF}^{\%M}$ +	-0.14	2.1×10^{-3}	0.41
	$\log_{10}(\%M)$	0.85	0.30	
Univariate models, excluding	$\log_{10}(S)$	1.46	0.11	0.05
escapes at the time the T cell	ΔE	-0.18	0.10	0.06
response was first detected	t_{WF}	-0.11	0.02	0.17
(n=49, maximum possible	$t_{WF}^{\%M}$	-0.15	4.3×10^{-3}	0.28
pseudo-R^2=0.69)	$\log_{10}(\%M)$	2.03	8.0×10^{-3}	0.18
Multivariate models, excluding	$\log_{10}(S)$ +	2.14	0.08	0.24
escapes at the time the T cell	$\log_{10}(\%M)$	2.28	8.2×10^{-3}	
response was first detected	ΔE +	-0.31	0.04	0.26
(n=49, maximum possible	$\log_{10}(\%M)$	2.32	3.1×10^{-3}	
pseudo-R^2=0.69)	t_{WF} +	-0.14	0.02	0.33
	$\log_{10}(\%M)$	2.23	7.7×10^{-3}	
	$t_{WF}^{\%M}$ +	-0.12	0.02	0.30
	$\log_{10}(\%M)$	1.07	0.22	

Analogous to Table 2, but with random, patient-specific baseline escape rates included in the CPH model. Contributions of vertical immunodominance (%*M*) and purely fitness-related measures (S, ΔE, t_{WF}) again are mostly independent. Note that here the maximum possible pseudo-R^2 is substantially lower than in Table 2.

Supplementary Table 4 | Criteria for selection of 8-11mer candidate epitopes from reactive 18mers that previously could not be reliably identified

Patient	HLA type	18-mer	Epitopes	Selection criteria
CAP45	A*23:01,04; A*29:02,03; B*15:10; B*45:01; Cw*16:01; Cw*16:02	PGPGVRYPLTFGWCFKLV	RYPLTFGW<br>RYPLTFGWCF	Known A*23:01<br>Known A*23:01
CH40	A*02:01; A*31:01; B*40:01; B*44:02; Cw*03:02; Cw*05:01	KELYPLASLRSLFGNDPS	KELYPLASL	Known A*02:01, B*40
CH77	A*02:05; A*02:05; B*53:01; B*57:01-04; Cw*04:01; Cw*18:01	LGLNKIVRMYSPTSILDI	RMYSPTSIL	Known A*02
CH77	A*02:05; A*02:05; B*53:01; B*57:01-04; Cw*04:01; Cw*18:01	FDSRLAFQHVAREIHPEF	VAREIHPEF	IC_{50}=649nM (<716nM, B*57:01-specific cutoff)
CH77	A*02:05; A*02:05; B*53:01; B*57:01-04; Cw*04:01; Cw*18:01	GKKQYKLKHIVWASRELE<br>+ HIVWASRELERFAVNPSL<br>+ LERFAVNPSLLETSEGCR	ASRELERF	IC_{50}=580nM (<716nM, B*57:01-specific cutoff)
CH164	A*02:01; A*29:01-04; B*44:03; B*45:01; Cw*07:01; Cw*16:01	KEGHIARNCKAPRKKGCW	KEGHIARNCKA	IC_{50}=428nM (B*45:01)
CH256	A*33:01; A*68:01; B*14:01; B*53:01; Cw*04:01; Cw*08:02	GQMVHQPLSPRTLNAWVK	MVHQPLSPR<br>QPLSPRTLNAW<br>QMVHQPLSPR	IC_{50}=19nM (A*68:01)<br>IC_{50}=42nM (B*53:01)<br>IC_{50}=298nM (A*33:01)

We attempted to infer likely 8-11mer epitopes for sets of reactive 18mers where the true epitopes had not previously been discerned experimentally (see Supplementary Ref. 4). The candidate epitopes identified above were selected based on the match to known epitopes in the LANL CTL database and to predicted epitope-HLA binding affinities. For details, see Methods.

Supplementary Table 5 | Correlation between true escape times and predictors when epitopes with escape mutations present at the time T cell responses were first detected are excluded, or with escape mutations reverted

	**Predictors**	**Pearson correlation**	***P* value**
Exclude epitopes with escape mutations at initial time (*n*=59)	*S*	-0.15	0.26
	ΔE	0.30	0.02
	t_{WF}	0.34	$9.3{\times}10^{-3}$
Exclude epitopes with escape mutations at initial time, include immunodominance (*n*=43)	*S*	-0.08	0.59
	ΔE	0.21	0.17
	t_{WF} + immunodominance	0.50	$6.6{\times}10^{-4}$
Revert escape mutations to T/F, include immunodominance (*n*=53)	*S*	-0.14	0.33
	ΔE	0.37	$6.4{\times}10^{-3}$
	t_{WF} + immunodominance	0.47	$3.4{\times}10^{-4}$

Escape occurs more rapidly when escape mutations are present in the virus population at the time that T cell responses are first detected. This is because the fitness cost of escape appears to be particularly low for these epitopes (Methods). Nonetheless, the correlation between fitness cost/time to escape in simulated evolution and the true escape time remains robust even if these epitopes are omitted.

Supplementary References

1. Leslie, A. J. *et al.* HIV evolution: CTL escape mutation and reversion after transmission. *Nat Med* **10,** 282–289 (2004).
2. Martinez-Picado, J. *et al.* Fitness Cost of Escape Mutations in p24 Gag in Association with Control of Human Immunodeficiency Virus Type 1. *J Virol* **80,** 3617–3623 (2006).
3. Mann, J. K. *et al.* The fitness landscape of HIV-1 Gag: Advanced modeling approaches and validation of model predictions by in vitro testing. *PLoS Comput Biol* **10,** e1003776 (2014).
4. Liu, M. K. P. *et al.* Vertical T cell immunodominance and epitope entropy determine HIV-1 escape. *J Clin Invest* **123,** 380–393 (2013).
